# New-Onset Crohn's Disease following Initiation of Secukinumab: A Case Report and Review of the Role of IL-17 in the Pathogenesis of Crohn's Disease

**DOI:** 10.1155/2023/1769290

**Published:** 2023-05-22

**Authors:** Anas Khouri, Cesar Moreno, Benjamin Niland

**Affiliations:** ^1^Department of Internal Medicine, University of South Alabama, Mobile, AL, USA; ^2^Division of Gastroenterology, University of South Alabama, Mobile, AL, USA

## Abstract

Patients with autoimmune conditions show a high expression of proinflammatory cytokines including interleukin (IL)-17. While IL-17 inhibitors have demonstrated efficacy in managing autoimmune disorders, rare instances of de novo or exacerbated inflammatory bowel disease (IBD) have been reported. The factors that affect the onset and severity remain unclear. Here, we present a case of a 38-year-old female who developed manifestations of Crohn's disease within 1 month of initiating secukinumab treatment for psoriatic arthritis, in addition to a review of the role of IL-17 in the pathophysiology of Crohn's disease.

## 1. Introduction

Interleukin-17 (IL-17), a proinflammatory cytokine, plays a significant role in the pathogenesis of autoimmune disorders such as psoriasis, psoriatic arthritis, and ankylosing spondylitis. Interestingly, IL-17 may play a protective role against inflammatory bowel diseases (IBDs) [1]. Secukinumab, a monoclonal antibody targeting IL-17, is used in the treatment of rheumatologic conditions. Although the occurrence of de novo IBD in patients receiving Secukinumab is very rare, with only 0.56% of patients experiencing it based on a pooled analysis of 21 clinical trials [2], the clinical significance of this infrequent event is unclear as multiple studies have produced mixed results [3]. Moreover, the identification of risk factors or genetic predispositions that could predict the development and severity of IBD in patients receiving Secukinumab is currently lacking.

## 2. Case Report

**Figure 1:**
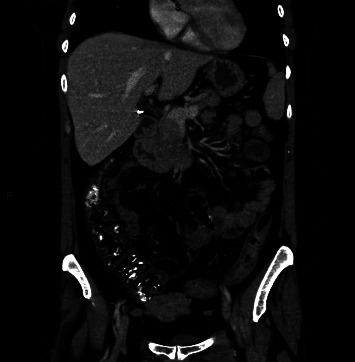
CT showed diffuse colitis.

A 38-year-old female with a history of psoriatic arthritis presented to the clinic complaining of generalized abdominal pain and diarrhea. One month prior to her presentation, she had initiated treatment with secukinumab loading dose 150 mg subcutaneous weekly. After receiving four doses of secukinumab, she developed fecal urgency and watery diarrhea, experiencing approximately 5 bowel movements daily, with intermittent hematochezia. In addition, she reported dyspepsia, nausea, fatigue, oral aphthous ulcerations, odynophagia, and a weight loss of 12 pounds over the course of one month. The patient had no prior personal or family history of IBD. The physical exam showed psoriatic lesions on the bilateral elbows and multiple inflamed ulcers on the posterior pharynx. The patient's abdomen was soft to palpation with tenderness in the periumbilical and lower abdominal regions. Laboratory evaluation revealed anemia and elevated inflammatory markers, including hemoglobin/hematocrit levels of 9.2/28.7, an erythrocyte sedimentation rate (ESR) of 40 mm/hr, and a C-reactive protein (CRP) level of 3.6 mg/dl. Computed tomography of the abdomen showed diffuse colitis (Figure 1).

**Figure 2:**
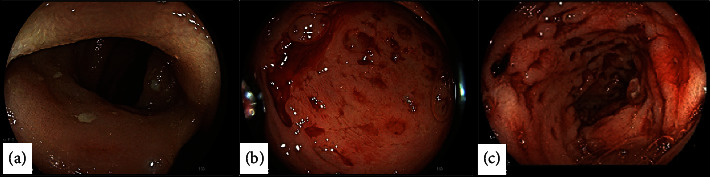
Colonoscopy on presentation shows terminal ileum with aphthous ulcerations (a) and scattered ulcerations in the rectosigmoid (b, c).

Secukinumab was discontinued, and the patient underwent esophagogastroduodenoscopy (EGD) and colonoscopy (CSC) to investigate the underlying pathology. EGD revealed normal esophagus, while ulcers were observed in the antrum along with duodenitis. CSC unveiled small ulcerations throughout the entire lumen of the terminal ileum and the cecum. The ascending and transverse colon appeared normal, but bleeding ulcerations, exhibiting both fresh and clotted blood, were observed in the descending colon, sigmoid colon, and rectum (Figure 2). Double balloon enteroscopy or capsule endoscopy was not performed to evaluate the extent of the disease in the jejunum.

**Figure 3:**
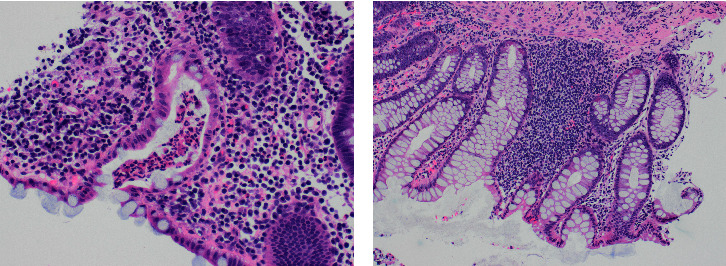
(a) Crypt abscess (active colitis) in the cecal biopsy specimen. (b) Paneth cell metaplasia in rectal biopsy indicating the ongoing inflammatory process.

Histopathological analysis of cecal biopsies revealed minimal architecture distortion in the large bowel mucosa, along with focal acute colitis. Similarly, biopsies obtained from the large bowel and rectum showed evidence of mildly active chronic colitis, with no evidence of dysplasia (Figure 3).

**Figure 4:**
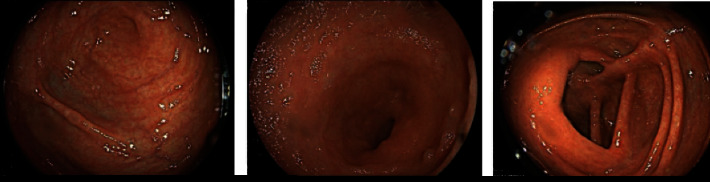
Colonoscopy after 6 months shows normal ileum and colon.

The diagnosis of ileocolitis Crohn's disease was made based on the previous findings, with Crohn's disease activity index (CDAI) of 284 points. Treatment was initiated with prednisone at a daily dosage of 40 mg, and secukinumab was switched to infliximab, resulting in the resolution of the patient's symptoms. Six months later, follow-up colonoscopy showed no active inflammation (Figure 4), and the fecal calprotectin level was <5 mcg/g. The CDAI score significantly decreased from 284 points at presentation to 3 points, indicating a substantial improvement in disease activity.

## 3. Discussion

The diagnosis of Crohn's disease typically requires a comprehensive and multifactorial approach, including clinical symptoms, laboratory tests, imaging studies, endoscopy, biopsy, and the exclusion of alternative infectious etiologies. In this case, we diagnosed our patient based on the presence of classic features of Crohn's disease, including fecal urgency, diarrhea, hematochezia, fatigue, abdominal pain, weight loss, and oral aphthous ulceration. Laboratory tests revealed anemia and elevated inflammatory markers, specifically ESR and CRP, indicating ongoing systemic inflammation which supported the diagnosis of Crohn's disease. A CT scan demonstrated ileitis and colitis. Endoscopic evaluation with biopsies revealed segmental ulcerations throughout the entire lumen of the terminal ileum and colon with pathologic evidence of inflammation. Collectively, these various criteria in conjunction with response to the steroid and infliximab therapy enabled us to confidently diagnose the patient with ileocolitis Crohn's disease.

The exact pathophysiology underlying inflammatory bowel disease remains unclear, with factors such as genetics, infections, medications, dietary factors, and immune responses implicated in the development of IBD and IBD-like lesions [4]. Extensive research has focused on understanding the role of the immune system and its inflammatory mediators in IBD, aiming to expand our treatment options by targeting specific cytokines involved.

IL-17 is a proinflammatory cytokine secreted by T-helper 17 (Th17) cells. Alongside other inflammatory mediators like tumor necrosis factor (TNF), IL-17 activates neutrophils and augments inflammatory responses. In normal circumstances, this activation serves to protect the intestinal mucosa against pathogens. However, dysregulated activation of IL-17 can lead to exaggerated inflammatory responses and the development of autoimmune diseases [5].

Th17 cells, a subset of CD4+ cells involved in cellular immunity, play a significant role in immune-mediated diseases by modulating inflammatory responses. These cells are activated by IL-23, another proinflammatory cytokine secreted by antigen-presenting cells (APCs).

IL-17 promotes inflammation within the bowel by inducing epithelial cells to secrete chemokines, which attract inflammatory cells to the site of inflammation. In addition, IL-17 stimulates myofibroblasts to release metalloproteinases, leading to damage in the extracellular matrix of epithelial cells [5].

The levels of IL-17 and IL-23 have shown a correlation with the presence and severity of IBD, suggesting their potential as biomarkers for the disease. Patients with severe ulcerative colitis and Crohn's disease with intestinal complications exhibit significantly higher levels of these interleukins than those with mild IBD, who, in turn, have higher levels than healthy individuals [6].

Therefore, medications targeting the IL-23/Th17/IL-17 pathway are used in the treatment of autoimmune conditions, such as psoriasis, psoriatic arthritis, ankylosing spondylitis, and IBD. Agents such as IL-23/IL-12 inhibitors (e.g., ustekinumab), IL-23 inhibitors (e.g., risankizumab), and TNF*α* inhibitors (e.g., infliximab and adalimumab) have demonstrated success in managing these conditions. However, IL-17 inhibitors (e.g., secukinumab and ixekizumab) have shown paradoxical effects in IBD.

The role of IL-17 in the context of inflammatory bowel disease (IBD) is complex and not yet fully understood. The IL-17 cytokine family consists of six different cytokines (IL-17A to IL-17F) and five IL-17 receptors (IL-17RA to IL-17RE). Studies using mice models have revealed distinct actions of these subtypes on the intestinal mucosa. For instance, mice deficient in IL-17A and IL-17RA exhibited more severe colitis, suggesting a protective role of IL-17A against acute colitis. Conversely, IL-17F deficient mice showed resistance to colitis, implying a pathogenic effect of IL-17F on the gut [5].

Secukinumab and ixecizumab are humanized IgG monoclonal antibodies that selectively bind to IL-17A. This could partially explain the worsening of IBD by blocking the protective effects of IL17A on the gut. It is not entirely clear why IL-17A, which is a proinflammatory cytokine in general, has a protective role on the gut, while it is proinflammatory in the joints. Additional studies are needed to explore these paradoxical effects of IL-17A.

IL-17 inhibitors have been associated with cases of IBD exacerbations, as well as de novo IBD cases. The incidence of new-onset IBD following the initiation of secukinumab is estimated to be around 0.2% to 1.3%. Gastrointestinal adverse events associated with IL-17 inhibitors occur in approximately 7.8% of cases. Majority of these events happen within the first year of treatment [7]. The severity of IBD associated with IL-17 inhibitors can vary widely, ranging from mild IBD-like lesions to rapid-onset fulminant colitis [8–10]. Distinguishing definite IBD from IBD mimickers, such as immune-mediated colitis, drug-induced colitis, and lymphocytic enteritis, can sometimes be challenging. These conditions can present with similar endoscopic and histological features as IBD and may be associated with monoclonal antibodies and immune-mediated therapies. Clinical factors, the timing of disease onset, and the presence of other immune-related adverse events can aid in differentiating between these entities [11].

Predicting how the gastrointestinal system will respond to IL-17 inhibitors remains a challenge. Until further studies establish a reliable model to risk stratify and predict the patients at risk of developing IBD, the severity, and the timing of onset based on risk factors or genetics, we suggest that clinicians consider alternative treatment options in patients with known IBD. In addition, we need to closely monitor for new-onset or worsening IBD following the initiation of IL-17 inhibitor therapy.
